# Detection of Antimalarial Resistance-Associated Mutations in Plasmodium falciparum via a Platform of Allele-Specific PCR Combined with a Gold Nanoparticle-Based Lateral Flow Assay

**DOI:** 10.1128/spectrum.02535-22

**Published:** 2022-11-29

**Authors:** Weijia Cheng, Wei Wang, Huiyin Zhu, Xiaonan Song, Kai Wu, Jian Li

**Affiliations:** a School of Basic Medical Sciences, Hubei University of Medicine, Shiyan, China; b Key Laboratory of National Health Commission on Parasitic Disease Prevention and Control, Jiangsu Provincial Key Laboratory on Parasites and Vector Control Technology, Jiangsu Institute of Parasitic Diseases, Wuxi, China; c Department of Clinical Laboratory, Wuchang Hospital Affiliated to Wuhan University of Science and Technology, Wuhan, China; d Department of Schistosomiasis and Endemic Diseases, Wuhan City Center for Disease Prevention and Control, Wuhan, China; University of Virginia

**Keywords:** malaria, mutation, Pfmdr1, *Plasmodium falciparum*, antimalarial drug resistance

## Abstract

Since single nucleotide polymorphisms (SNPs) have attracted attention, there have been many explorations and improvements in screening and detection methods for SNPs. Traditional methods are complex and time-consuming and rely on expensive instruments. Therefore, there is an urgent need for a low-cost, simple, and accurate method that is convenient for use in resource-poor areas. Thus, a platform based on allele-specific PCR (AS-PCR) and a gold nanoparticle-based lateral flow assay (LFA) was developed, optimized, and used to detect the SNPs of the drug resistance gene *pfmdr1*. Subsequently, the system was assessed on clinical isolates and compared with nested PCR followed by Sanger sequencing. The sensitivity and specificity of the AS-PCR-LFA platform were up to 99.43% and 100%, respectively, based on the clinical isolates. The limit of detection is approximately 150 fg/μL for plasmid DNA as the template and 50 parasites/μL for dried filter blood spots from clinical isolates. The established and optimized AS-PCR-LFA system is more adaptable and rapidly translated to SNP analysis of other drug resistance genes and genetic diseases. In addition, while actively responding to the point-of-care testing policy, it also contributes to the Global Malaria Eradication Program.

**IMPORTANCE** Rapid detection of single nucleotide polymorphisms (SNPs) is essential for malaria treatment. Based on the techniques of allele-specific PCR (AS-PCR) and lateral flow assay (LFA), an accurate and powerful platform for SNP detection of *pfmdr1* was developed and evaluated with plasmid and clinical isolates. It offers a useful tool to identify antimalarial drug resistance and can support the effort to eliminate malaria globally.

## INTRODUCTION

Single nucleotide polymorphisms (SNPs) (1) are DNA sequence polymorphisms caused by nucleotide mutations at the genomic level. SNPs in the genome are closely related to human health (2–4). Mutations in SNPs can not only interfere with the occurrence and development of various diseases but also serve as potential diagnostic or prognostic molecular markers (5–7). In particular, they play an increasingly significant role in the early diagnosis or prognostic monitoring of patient personalization and targeted therapy (7, 8). Therefore, the identification of SNPs is particularly important in disease diagnosis, biomedical research, food safety, and environmental analysis (9). Currently, PCR-restriction fragment length polymorphism (PCR-RFLP) (10), nested PCR (11), allele-specific PCR (AS-PCR) (6), real-time fluorescence quantitative PCR (qPCR) (12), loop-mediated isothermal amplification (LAMP) (13), Sanger sequencing (11, 14), and gene chips (15) are commonly used to detect known SNPs. For unknown SNPs, single-strand conformation polymorphism (SSCP) (16), random amplified polymorphic DNA (RAPD) (17), high-throughput sequencing (next-generation sequencing [NGS]) (18), single-molecule real-time (SMRT) sequencing technology (19), and nanopore sequencing technology (20) are commonly used. However, most of these technologies have various traits, such as cumbersome operation, heavy time and labor consumption, expensive equipment, requirements for professional knowledge and skill, and difficulty in automation, which make them difficult to widely use in clinical environments.

At present, the most widely used method is AS-PCR, which has the characteristics of simplicity and economy, but the significant weakness of this technique is a high rate of false positives (21). The whole amplification reaction is primer dependent rather than template dependent. It has been reported that high-fidelity enzymes can be used to replace low-fidelity enzymes to solve the problem of high rates of false positives in SNP analysis (6). In addition, it is generally believed that artificial introduction of mismatched bases in the primers can improve the method’s specificity (6, 22). In previous work, the 3′-terminal double phosphorothioate-modified primer was used to further improve the specificity of AS-PCR (6, 7).

Another common method is sequencing, which is currently the gold standard for testing gene sequences (11, 12, 14). However, the test cycle is long, and the steps are too complicated, so the results cannot be obtained in time and cannot quickly be used in medical treatment for critical patients. More importantly, sequencing brings substantial economic expenditure to resource-deficient regions. Therefore, there is an urgent need for a point-of-care testing (POCT) strategy-based method to quickly interpret the results. The lateral flow assay (LFA) (23) has been suggested as an effective analytical technique for POCT. The assay can be initiated by a simple test strip contact with the sample and does not require additional manipulations with reagents and devices. A quick immunospecific reaction leads to visually detectable stained zones in several regions of the test strip with nanoparticle-labeled immune complexes. The accuracy has been verified in preliminary work (6, 7). Therefore, further improvement is needed for SNP detection, and a new platform with high sensitivity and specificity, low cost, convenience, and universal applicability is still needed.

**FIG 1:**
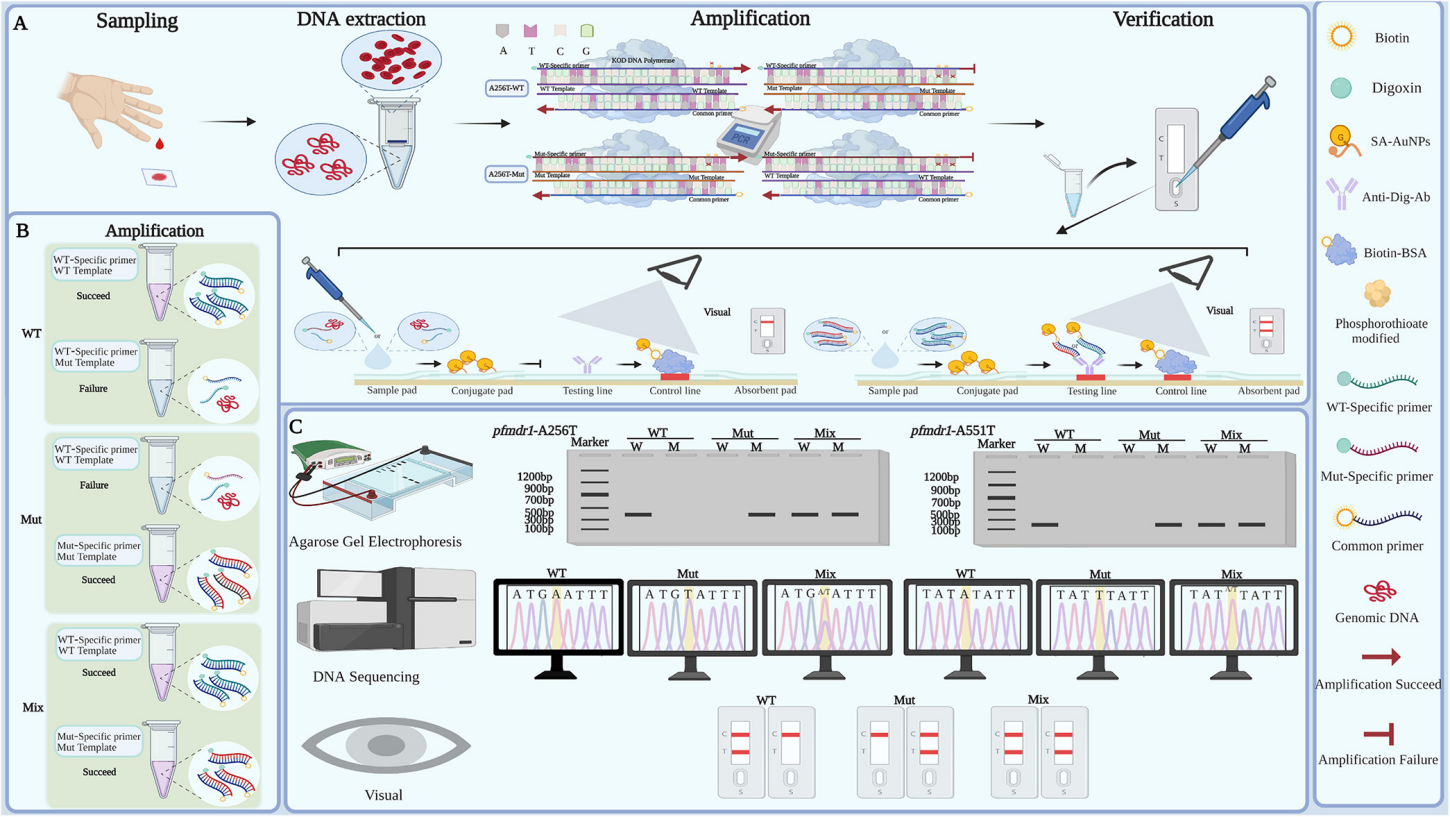
Schematic diagram of allele-specific PCR (AS-PCR) combined with a lateral flow assay (LFA) system for SNP detection. (A) The workflow of SNP site detection (taking *pfmdr1* A256T as an example). Genomic DNA (gDNA) was extracted from a dried filter blood spot (DBS) and used as the template for AS-PCR amplification. After amplification, the PCR products can be directly added to the strips, and they are interpreted according to whether the T line is colored. (B) The SNP detection relies on the wild-type-specific primers and mutant-type-specific primers. Briefly, the wild-type tube includes the wild-type template and primer, and the mutant-type tube includes the mutant-type template and primer. The amplification process will be blocked when the wild-type tube includes the mutant-type template and wild-type primer and when the mutant-type tube includes the wild-type template and mutant-type primer. Both tubes were amplified successfully in the presence of mixed type. WT, Mut, and Mix represent the wild type, mutant type, and mixed type, respectively. (C) Results of *pfmdr1* A256**T** and A551**T** were analyzed based on agarose gel electrophoresis, DNA sequencing, and signal readout by visual interpretation in LFA. W and M on the sample represent wild-type and mutant-type primers, respectively. C and T represent the control line and test line, respectively.

In this study, a method based on the AS-PCR-LFA platform for the rapid detection of SNPs with high sensitivity and specificity and relative rapidity and of general type was established and assessed (Fig. 1). With Plasmodium falciparum multidrug resistance gene 1 (*pfmdr1*) (2) as the target gene, the optimized system achieves high sensitivity and specificity, which are suitable for the detection of any SNPs. In practical applications, this study verified the stability and accuracy of the AS-PCR-LFA rapid detection method by comparing the detection method with the data from Sanger sequencing. The established platform not only has wide application value in resource-poor areas and clinical research but also offers a feasible method for molecular diagnosis.

## RESULTS

### Sensitivity and specificity validation of AS-PCR-LFA.

**FIG 2:**
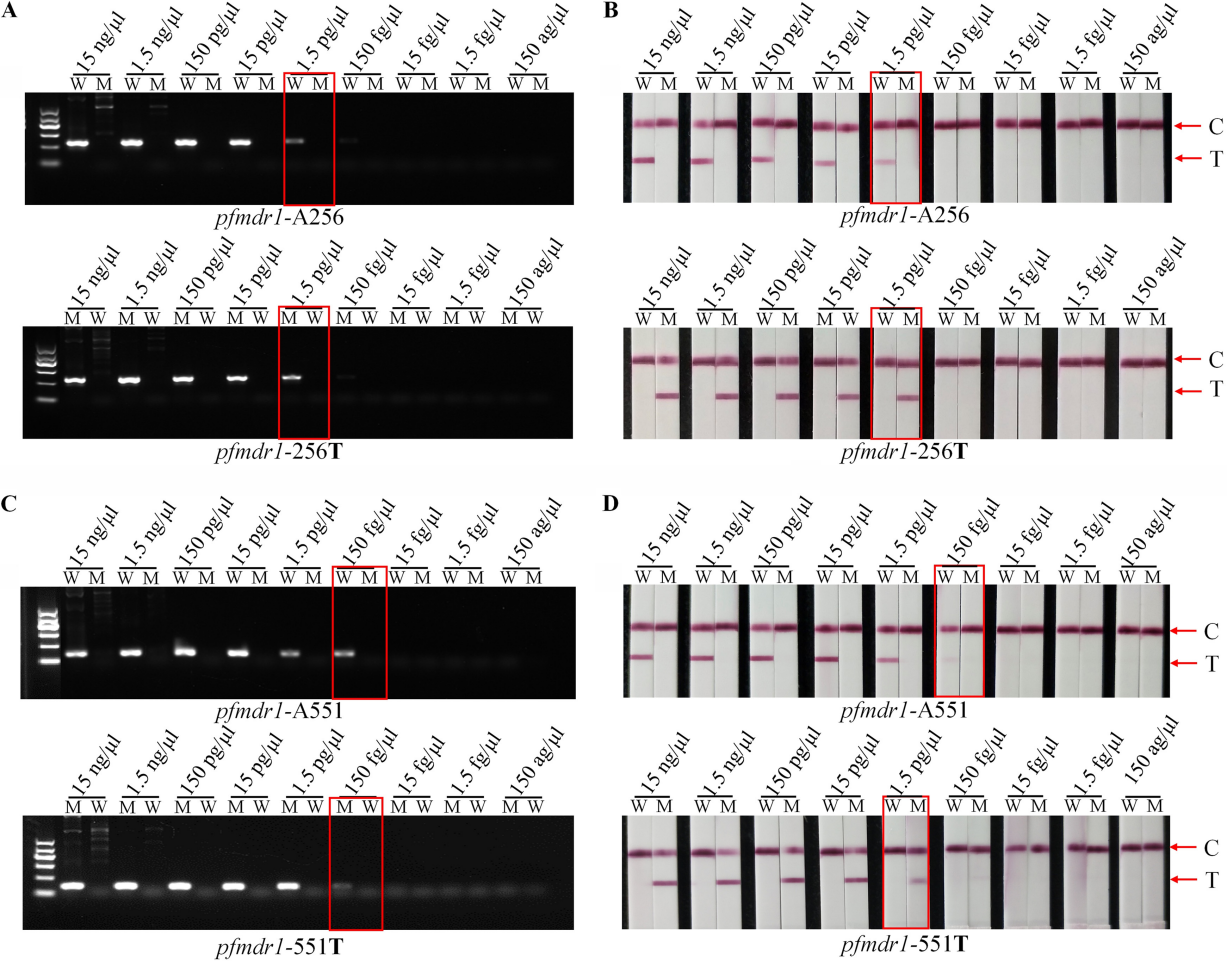
AS-PCR-LFA sensitivity and specificity validation of A256T and A551T. (A) Different concentrations of plasmid by agarose gel electrophoresis. (B) Different concentrations of plasmid by LFA. (C) Different concentrations of plasmid by agarose gel electrophoresis. (D) Different concentrations of plasmid by LFA. M indicates the DNA molecular markers, including 100 bp, 300 bp, 500 bp, 700 bp (blod), 900 bp, and 1,200 bp. C and T represent the control line and test line, respectively.

**FIG 3:**
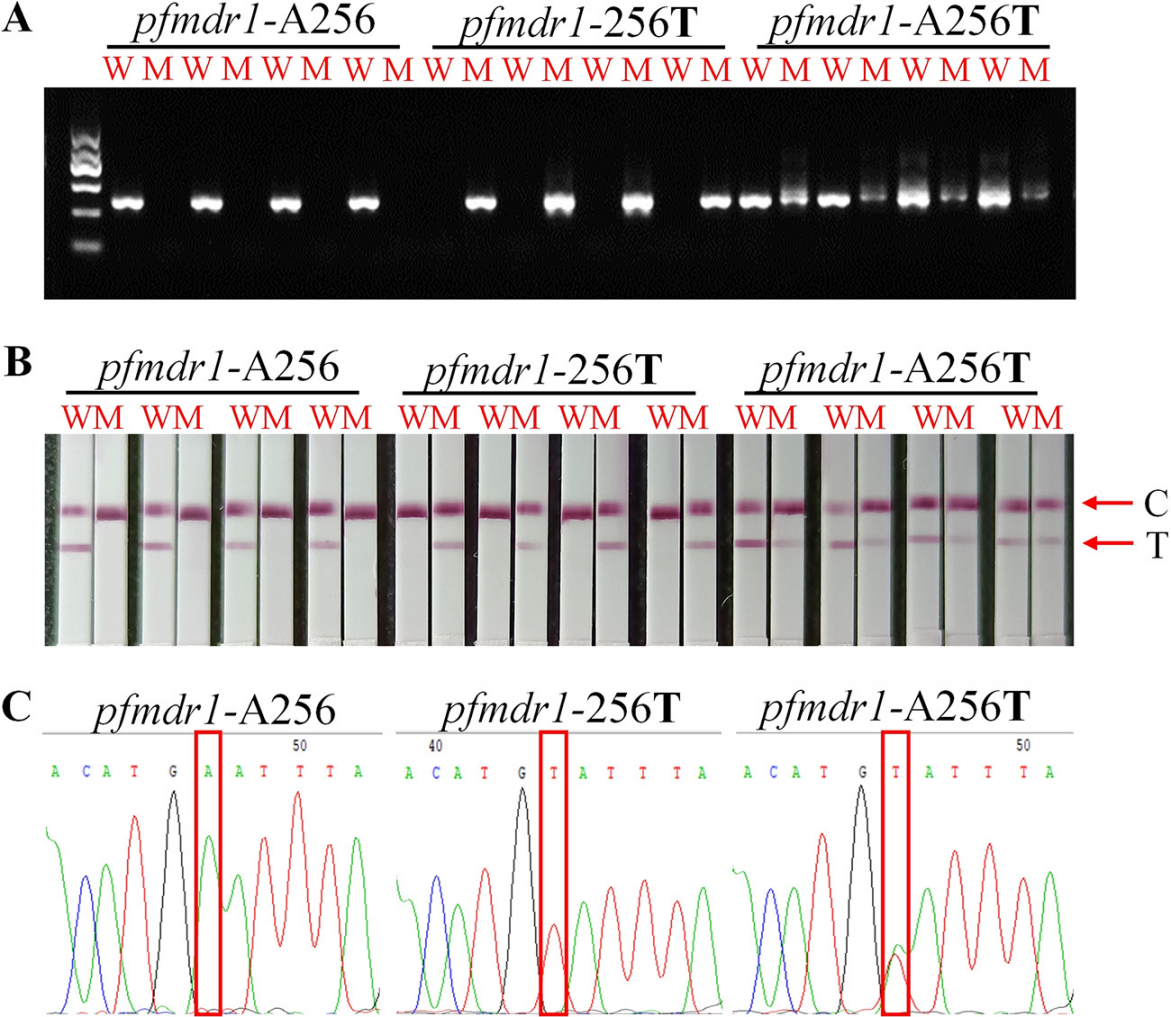
Genotyping result (taking partial samples as an example) of A256T. (A) Agarose gel electrophoresis; (B) AS-PCR-LFA system through visualized interpretation; (C) DNA sequencing. M indicates the DNA molecular markers, including 100 bp, 300 bp, 500 bp, 700 bp (blod), 900 bp, and 1,200 bp. C and T represent the control line and test line, respectively.

Based on the optimized reaction system and conditions (the detailed optimization is shown in the supplemental material), the sensitivity and specificity of AS-PCR were assessed. The plasmids were diluted according to different gradients and used as the templates. The final concentration of plasmids was set from 15 ng/μL to 150 ag/μL (Fig. 2 and 3). The optimized PCR system was used. A 25-μL final volume contained 1.0 μL of 10 μM common antisense primer, 2.5 μL 10× KOD buffer, 0.5 μL of 0.5 U of KOD-Plus-Neo, 2.5 μL of 2 mΜ deoxynucleoside triphosphates (dNTPs), 1.5 μL of 25 mM MgSO_4_, 1.0 μL of 10 μM allele-specific primer, and 1.0 μL of plasmid DNA (pDNA). The AS-PCR program for different loci is shown in Table S2 in the supplemental material. On 1.5% agarose gel electrophoresis, the limits of detection (LODs) of both SNP A256 and SNP 256**T** were 1.5 pg/μL (Fig. 2A and B). For A551, the concentration was 150 fg/μL (Fig. 2C and D). The limit for 551**T** in electrophoresis was 150 fg/μL, and that in LFA was 1.5 pg/μL (Fig. 2C and D). For specificity, wild-type primers amplified the wild type, and the mutant type was not amplified (Fig. 2). The results for mutant-type primers were the same, and the amplification band was single without a crossover reaction (Fig. 2). The results showed that the specificity of the optimized platform for SNP typing of the *pfmdr1* gene was 100%. For LFA, the band signal was consistent with the agarose gel electrophoresis results (Fig. 2).

### Evaluation of clinical application.

**FIG 4:**
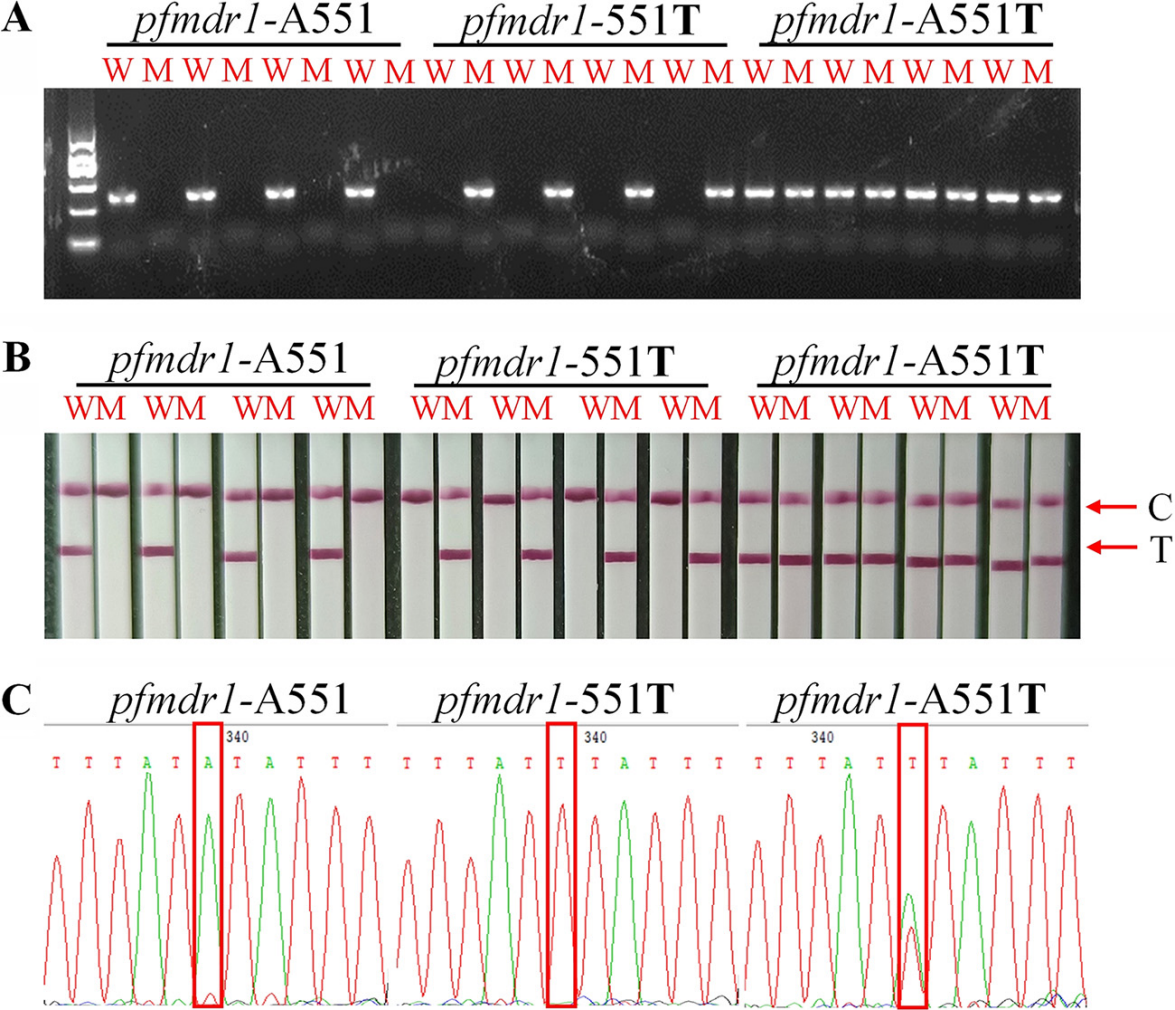
Genotyping result (taking partial samples as an example) of A551T. (A) Agarose gel electrophoresis; (B) AS-PCR-LFA system through visualized interpretation; (C) DNA sequencing. M indicates the DNA molecular markers, including 100 bp, 300 bp, 500 bp, 700 bp (blod), 900 bp, and 1,200 bp. C and T represent the control line and test line, respectively.

To test the dependability of the optimized AS-PCR-LFA system, the results were further confirmed on clinical samples. The ultimate wild-type and mutant-type results for each SNP were visually explained by the presence or absence of the color of the T line on the LFA. According to the results, the wild-type primer A256**T** successfully amplified the wild-type strain, and the target fragment was obtained by electrophoresis (Fig. 3A). The T line was obtained on the wild-type LFA (Fig. 3B). Similarly, mutant-type primers successfully amplified only mutants of the *pfmdr1* gene (Fig. 3A), and the T line also displayed color only on the mutant-type LFA (Fig. 3B). Two bands were obtained from the amplified mixed type by electrophoresis (Fig. 3A). Similarly, the T line was colored on both wild-type and mutant LFAs (Fig. 3B). All the results of DNA electrophoresis and LFA were compared with the data from Sanger sequencing (Fig. 3C). Subsequently, A551**T** was verified by the same procedure. The results showed that the wild-type and mutant types were successfully distinguished, and the results of electrophoresis, LFA, and sequencing were consistent (Fig. 4).

The clinical samples were genotyped by nested PCR and sequencing. Subsequently, AS-PCR-LFA system detection was performed, and the results are shown in Table S3. The methodological evaluation and analysis of the AS-PCR-LFA results and nested PCR with sequencing results are presented in Table 1. The sensitivity, specificity, false-negative rate, and false-positive rate of AS-PCR-LFA for A256**T** were 98.92%, 100%, 0.36%, and 0.00%, respectively. For A551**T**, they were 95.67%, 100%, 4.33%, and 0.00%, respectively. Finally, the LOD of AS-PCR-LFA was 50 parasites/μL of dried filter blood spot (DBS) in 277 clinical samples.

**TABLE 1:** Methodological comparison of the AS-PCR-LFA platform and nested PCR with sequencing in different levels of parasitemia

Genotype and parasitemia (parasites/μL)	Method				Sensitivity (%)	Specificity (%)	False negative (%)	False positive (%)
	Sequencing, no.	AS-PCR-LFA^*a*^						
		WT	Mut	Mix				
A256T								
Low (≤1,000)	53	44	6	2	98.11	100	1.89	0
Medium (1,001–9,999)	49	42	6	0	97.96	100	2.04	0
High (≥10,000)	175	145	28	1	99.43	100	0.57	0
								
Subtotal	277	231	40	3	98.92	100	0.36	0
A551T								
Low (≤1,000)	53	15	18	15	90.57	100	9.43	0
Medium (1,001–9,999)	49	17	27	4	97.96	100	2.04	0
High (≥10,000)	175	66	90	13	96.57	100	2.86	0
								
Subtotal	277	98	135	32	95.67	100	4.33	0

a WT, Mut, and Mix represent the wild type, mutation, and mixed type, respectively.

## DISCUSSION

Modern biotechnology is faced with how to detect known SNPs quickly and reliably. In recent years, the problem of antimalarial drug resistance of P. falciparum has become severe and will hinder the global malaria eradication plan (11, 24). If patients are infected by drug-resistant P. falciparum parasites, antimalarial drugs will be no longer effective. Falciparum malaria patients will progress to severe malaria, particularly cerebral malaria (25), and may easily die if they are not treated promptly. Thus, rapid drug resistance monitoring is particularly important. To solve this problem, this method was established.

Since SNPs began to receive attention, there have been many explorations and improvements in screening and detection methods (7, 26). At present, nested PCR with sequencing is considered the gold standard for SNP detection (11). However, it takes a long time and requires a professional to report the results. The qPCR is also often used for SNP detection of drug resistance genes, and it has the advantages of being fast and having high specificity (12). Because the equipment is expensive, it cannot be tested in the field, nor can it be widely promoted in resource-poor areas. LAMP has been used for SNP typing and has the advantage of being able to amplify at a single temperature (27). However, factors such as high false-positive rates and difficult primer designs keep it from being widely promoted. For epidemiological surveillance, it becomes expensive in terms of both equipment and operating costs. In addition, AS-PCR is also widely used as the most common and easiest detection method. However, due to the limitations of the method, nonspecific amplification often occurs (28). Therefore, there is an urgent need for a low-cost, rapid, and accurate detection system that can be used for high-throughput detection of SNPs in drug resistance genes. In one study (27), LAMP combined with the LFA method was established based on the N51 Isoleucine (**I**) mutation site of the *pfdhfr* gene, but the detection limit was only 2 ng/μL. In our previous work, the LOD of N51**I** in the *pfdhfr* gene was 20 fg/μL by the AS-PCR-LFA system initially established (6). Subsequently, the rapid detection method was optimized and applied to the *pfcrt* gene (7), and the LOD was 1.5 pg/μL. To better verify the repeatability and stability of this rapid detection method and to further reduce the detection limit, in this study, the AS-PCR-LFA system was used to detect wild-type and mutant types at sites A256**T** and A551**T** in the *pfmdr1* gene. Compared to previously used techniques, the cost is significantly reduced, and large samples can be analyzed in a relatively short time. The technology can be transferred and operated in laboratories with minimal infrastructure.

In the AS-PCR improvement in this study, each specific primer utilized a 3′-terminal artificial mismatch and was then double phosphorothioate modified and combined with KOD DNA polymerase. The false-positivity problem of traditional AS-PCR was solved fundamentally. In the system optimization, avoiding a high annealing temperature affects the sensitivity of subsequent experiments. For A256, A551, and 551**T**, 61.1°C was selected, while for 256**T**, 60.5°C was selected for annealing temperature. Finally, the SNP detection results were interpreted according to the color change of the T lines on the LFA. The AS-PCR-LFA system proved to be fast and accurate. In clinical samples, the specificity was 100%. The sensitivity of clinical samples with high parasitemia at A256**T** was up to 99.43%. Moreover, the pDNA was detectable at 150 fg/μL, and in clinical samples, the LOD was 50 parasites/μL of DBS. The most critical factor for this technique seems to be the quality of genomic DNA (gDNA) in clinical samples. The complex composition of gDNA extracted from DBSs and the variety of PCR inhibitors greatly affect the amplification efficiency. In addition, P. falciparum is particularly difficult to amplify and sequence. The AT content of intergene regions and introns in P. falciparum exceeds 80%, and there are a large number of AT-rich repeats (29). Therefore, part of the sample in this study was not successfully amplified. Finally, the improved and optimized AS-PCR-LFA has high specificity and sensitivity, allowing large-scale SNP screening in a very short period. Especially in resource-poor areas, such as Africa, and in field testing, only low-cost, highly accurate methods can effectively conduct mass screening.

Based on the shortcomings of this study, the system can be further improved. Based on the POCT strategy, a simplified version of the gDNA extraction method is used to replace the existing scheme while ensuring the quality of gDNA. The present constant thermostatic amplification technique, especially recombinase polymerase amplification (30), can be used to shorten the amplification time and simplify the operation. Alternatively, the introduction of CRISPR/Cas12a (22) will further improve the sensitivity and specificity of the method.

In summary, the established AS-PCR-LFA detection platform can be used to conduct large-scale screening in a short time. The cost per SNP process is significantly reduced compared to other methods, and the platform can also be widely used for SNP analysis of other drug resistance genes. Meanwhile, it also provides convenience for guiding clinical medication, personalized treatment, and POCT strategies.

## MATERIALS AND METHODS

### Materials and reagents.

All reagents used were analytical reagents. PCR was performed using KOD-Plus-Neo from Toyobo Co., Ltd. (Shanghai, China), and Phanta Max master mix from Vazyme Biotech Co., Ltd. (Nanjing, China). Streptavidin-immobilized gold nanoparticles (SA-AuNPs) were obtained from Beijing Biosynthesis Biotechnology Co., Ltd. (Beijing, China). Biotinylated bovine serum albumin (biotin-BSA) was acquired from Solarbio (Beijing, China). The consumables required to test the strip system were from Shanghai Kinbio Tech. Co., Ltd. (Shanghai, China).

### Principle of the AS-PCR-LFA system.

To detect SNPs at sites A256**T** and A551**T** of the *pfmdr1* gene with sensitivity and specificity, an opportunistic AS-PCR-LFA detection system was developed (Fig. 1). Taking A256**T** as an example, the quick detection process is shown in Fig. 1A. First, the dried filter blood spot (DBS) is made from fresh blood of patients, and genomic DNA (gDNA) is extracted from DBS and then amplified by AS-PCR. Additional base mismatches and phosphorothioate modifications are designed to increase the specificity of the amplification. After amplification, the PCR products can be directly added to the strips, and the results are interpreted according to whether the T line is colored (Fig. 1A). The biotin labels on the 5′ end of allele-specific primers in PCR products are identified and captured by SA-AuNPs on the conjugate pad and driven by capillary force. When the target products are present, the digoxigenin (Dig) labels on the 5′ ends of common primers are bound and aggregated by an immobilized antidigoxigenin monoclonal antibody (Ab) that is fixed on the T line. Because of the presence of AS-AuNPs, the T line first presents a gold-red band. The remaining SA-AuNPs continue forward and are then captured by biotin-BSA on the C line, presenting another red band. If there are no target products, there is no red band on the T line. Genotyping is shown in Fig. 1B. When the wild-type or mutant primers bind to the corresponding bases of the wild type or mutant, amplification can proceed smoothly. The wild-type tube includes the wild-type template and primer, and the mutant-type tube includes the mutant-type template and primer. The amplification process will be blocked when the wild-type tube includes the mutant-type template and wild-type primer and when the mutant-type tube includes the wild-type template and mutant-type primer. Both tubes are amplified successfully in the presence of mixed type (Fig. 1B). The genotyping results can be interpreted by three methods (Fig. 1C). They can be visually interpreted by LFA in less than 10 min and without additional equipment, replacing traditional DNA sequencing.

### Plasmid construction and identification.

The sequence of the *pfmdr1* gene (PF3D7_0523000) from the P. falciparum 3D7 strain was downloaded from the PlasmoDB database (https://plasmodb.org/plasmo/app). The truncated fragments (572 bp) of the *pfmdr1* gene with different SNPs were synthesized by Genewiz Biotechnology Ltd. (Soochow, China). Subsequently, the two target sequences were cloned into the vector pUC57 to generate the recombinant plasmids pUC57/*pfmdr1*-A256-A551 (wild type) and pUC57/*pfmdr1*-256**T**-551**T** (mutant type). Subsequently, they were validated through restriction digestion with BamHI and XhoI (see Fig. S1A in the supplemental material). Two fragments of approximately 3,357 bp and 571 bp were detected, both consistent with the vector and the target fragment (Fig. S1A). Finally, the plasmids were sequenced (Fig. S1B) and were identical to the matched target sequence, indicating that they were successfully constructed. Finally, concentration and quality were evaluated by Gene5 (Thermo Fisher Scientific, Wilmington, DE, USA).

### Design and screening of oligonucleotides.

The rules of allele-specific primer design proposed in a previous work were followed (6), with slight modifications. Briefly, each specific primer utilized a 3′-terminal artificial mismatch and double phosphorothioate modification. Therefore, the primer candidates with A256T are listed in Table S1. Four wild-type allele-specific primers, three mutant-type allele-specific primers, and one universal primer were designed. Similarly, the same design was used for A551T, as shown in Table S1, and Oligo 7 software was used for evaluation.

For A256 (Fig. S2A), the wild-type allele-specific primer named W-F0 appeared false positive during amplification when using the mutant template. The amplification efficiency of W-F1 was lower than that of W-F2 and W-F3. The bands of W-F2 were clearer, so it was selected as the most specific primer. For 256**T** of the most specific primer screening (Fig. S2B), M-F1 had the lowest amplification efficiency. M-F2 can still amplify a single band at 58.4°C. Therefore, it was selected as the optimal primer. Screening was continued for the A551 and 551**T** primers under the same conditions. For A551 (Fig. S3A), all the primers had specificity; W-F0 and W-F2 could still be successfully amplified at different annealing temperatures, but W-F2 had a slightly better effect than W-F0, so W-F2 was selected as the best primer for A551. Among the specific primers designed for 551**T** (Fig. S3B), M-F3 has the worst sensitivity, while M-F1 and M-F2 both have specificity. M-F2 showed the best performance at different annealing temperatures.

### Establishment and optimization of the AS-PCR-LFA platform.

The annealing temperature, cycle numbers, concentrations of MgSO_4_, and primers in the reaction system significantly influence the amplification efficiency. Thus, to improve the sensitivity and specificity of target gene amplification, PCR conditions were optimized (see the supplemental material). The plasmid was diluted to 1:10 and used as the template for system optimization. The LFA (Fig. 1A) (width, 4 mm) consists of five components, including a sample pad (17 mm in length), conjugate pad (8 mm), NC membrane (25 mm), and absorbent pad (17 mm), which were then fixed to a viscous plastic backing with a 2-mm overlap. Subsequently, the LFA was soaked in a pretreatment solution for 30 min and then dried overnight at 56°C. Next, biotin-BSA and anti-Dig antibody (Ab) were fixed on the control line and test line, respectively, using the XYZ platform dispenser (model HM3035). In addition, the solution containing SA-AuNPs was fixed on the bonding pad. Then, they were dried again at 56°C for 4 h. On the LFA, 2.0-μL PCR products were dripped onto the sample pad, followed by 70 μL buffer (pH 7.4). The results can be determined in approximately 10 min by the presence of red lines (Fig. 1C). Similarly, the results of SNPs can be analyzed by agarose gel electrophoresis and sequencing (Fig. 1C).

### Sensitivity and specificity validation of the AS-PCR-LFA platform.

The plasmids pUC57/Pfmdr1_NY_ and pUC57/Pfmdr1**_YF_** were diluted according to different gradients and used as the template. For AS-PCR, the final concentrations of plasmid were from 150 ag/μL to 15 ng/μL. The reaction was carried out under optimum conditions. The qualitative and quantitative detection limits (LODs) were defined as the plasmid concentration corresponding to the lightest color visible to the naked eye. The LOD was used as the standard for sensitivity evaluation. Meanwhile, the plasmids pUC57/Pfmdr1_NY_ and pUC57/Pfmdr1**_YF_** were cross amplified with wild-type allele-specific primers and mutant-type allele-specific primers. The specificity was evaluated by observing whether there was a specific amplification strip.

### Assessment of the AS-PCR-LFA platform with clinical isolates.

In preliminary work (11), peripheral venous blood from patients infected with P. falciparum parasites was successfully collected, and DBSs were made. After nucleic acid was extracted from 277 clinical samples, SNPs were detected by the established rapid method, and these samples were detected by nested PCR with Sanger sequencing, which was used as a reference method. The consistency of the results was used to evaluate the clinical application.
