# Supplementary material for: Detection of Antimalarial Resistance-Associated Mutations in Plasmodium falciparum via a Platform of Allele-Specific PCR Combined with a Gold Nanoparticle-Based Lateral Flow Assay
Source: Microbiol Spectr. 2022 Nov 29;10(6):e02535-22. doi: 10.1128/spectrum.02535-22 (PMC9769821; doi:10.1128/spectrum.02535-22)
Supplement: Supplemental file 1 — Supplemental text and Fig. S1 to S4. Download spectrum.02535-22-s0001.pdf, PDF file, 1.3 MB [file spectrum.02535-22-s0001.pdf]

Detection of antimalarial resistance-associated mutations in *Plasmodium falciparum* via a platform of allele-specific PCR combined with gold nanoparticles based lateral flow assay

Weijia Cheng[a,c,*], Wei Wang[b,*], Huiyin Zhu[a], Xiaonan Song[a], Kai Wu[d], Jian Li[a,#]

[a]School of Basic Medical Sciences, Hubei University of Medicine, Shiyan 442000, China
[b]Key Laboratory of National Health Commission on Parasitic Disease Prevention and Control, Jiangsu Provincial Key Laboratory on Parasites and Vector Control Technology, Jiangsu Institute of Parasitic Diseases, Wuxi 214064, China
[c]Department of Clinical Laboratory, Wuchang Hospital Affiliated to Wuhan University of Science and Technology, Wuhan, 430063, China
[d]Department of Schistosomiasis and Endemic Diseases, Wuhan City Center for Disease Prevention and Control, Wuhan 430024, China

Running Title: Mutation detection of *P. falciparum* by AS-PCR-LFA

* These authors contributed equally and the order of authorship is randomly assigned.

# Corresponding author
Email: yxlijian@163.com. Tel.: 86-719-8891141 (Jian Li)

Supporting information

Establishment and optimization of the AS-PCR-LFA platform

Optimization of annealing temperature

To determine the optimal annealing temperature. According to the Tm value of the primers, different annealing temperatures were set at 58, 58.2, 58.4, 58.9, 59.5, 60, 60.5, 61.1, 61.6, 62, 62.3, and 62.5 °C. For A256 and 256**T**, both amplify from bands of the same size as expected. For A256, when the annealing temperature rises to 60 °C, the specificity increases obviously, as shown in *pfmdr1*-A256-W-F2 (Fig. S2A). Therefore, 61.1 °C was selected as the best annealing temperature. For 256**T**, the specificity begins to improve significantly at 58.9 °C. However, at 61.6 °C, the amplification band disappeared, so 60.5 °C was selected as the optimal annealing temperature, as shown in *pfmdr1*-256**T**-M-F2 (Fig. S2B). For A551 and 551**T**, as shown in *pfmdr1*-A551-W-F2 (Fig. S3A) and *pfmdr1*-551**T**-M-F2 (Fig. S3B), a single target band could be amplified at different annealing temperatures, and the wild-type and mutant templates could be effectively differentiated. To facilitate amplification, 61.1 °C was also selected as the optimal annealing temperature.

Concentration optimization of $MgSO_4$

In $MgSO_4$ optimization, different concentrations of 0, 0.5, 1.0, 1.5, 2.0, 2.5, 3.0, and 3.5 mM were set for screening. For A256 and 256**T**, according to the results (Fig. S4A), when the $MgSO_4$ concentration is too low, there is no amplification band. At 1.0 mM, the bands became brighter, and nonspecific amplification was observed again at 2.0 mM. Therefore, 1.5 mM was chosen as the optimal concentration. Analogously, 1.5 mM was selected as the optimal $MgSO_4$ concentration for 551A and 551**T** (Fig. S4D).

Concentration optimization of primers

The primer concentration was optimized, and different concentrations of 0.04, 0.1, 0.2, 0.3, 0.4, 0.5, 0.6, and 0.7 μM were set for screening. According to the results of AS-PCR amplification (Fig. S4B and E), the target band was gradually enhanced with increasing primer concentration. The phenomenon of nonspecific amplification. For A256, 256**T**, 551A, and 551**T**, all at 0.4 μM, the bands were bright. After comprehensive consideration, the final concentration of their primers was determined to be 0.4 μM in subsequent experiments.

Optimization of cycles number

Finally, based on the above optimization conditions, we continue to optimize the number of cycles. There were 15, 20, 25, 30, and 35 cycles, respectively. For A256, 256**T** (Fig. S4C), and 551**T** (Fig. S4F), amplification bands appeared in 15 cycles and gradually increased with increasing cycle number. Therefore, choosing 30 cycles is the best. For A551 (Fig. S4F), weak bands appeared in the 15 cycles, and the amplification bands gradually increased with the increase in the number of cycles, while at 35 cycles, the bands began to weaken. To facilitate later amplification, 30 cycles were selected as the optimal number of cycles.

Fig. S1 Recombinant plasmid construction and identification. A: Double digestion with the restriction endonucleases *BamHI* and *XhoI*. M represents the DL5000 DNA ladder (100 bp, 250 bp, 500 bp, 750 bp, 1 kb, 2 kb, 3 kb, 4 kb, and 5 kb). pDNA represents plasmid DNA. Number of 1 and 2 represent the recombinant plasmids *pUC57-pfmdr1*-A256-A551 (wild-type) and *pUC57-pfmdr1*-256**T**-551**T** (mutant-type), respectively; **B**: The peak map of the constructed recombinant plasmid from Sanger sequencing.

Fig. S2 Primer screening for A**256T detection in the *pfmdr1* gene. A**: The distinguishing primer for wild-type at positions A256; **B**: The distinguishing primer for mutant-type at positions 256**T**. Number of 1–12 result from annealing temperatures of 58, 58.2, 58.4, 58.9, 59.5, 60, 60.5, 61.1, 61.6, 62, 62.3, and 62.5 °C, respectively. The M indicates the DNA molecular marker, including 100 bp, 300 bp, 500 bp, 700 bp (blod), 900 bp, and 1200 bp. C and T represent the control line and test line respectively.

Fig. S3 Primer screening for A**551T detection in the *pfmdr1* gene. A**: The distinguishing primer for wild-type at position A551; **B**: The distinguishing primer for mutant-type at position 551**T**. Number of 1–12 result from annealing temperatures of 58, 58.2, 58.4, 58.9, 59.5, 60, 60.5, 61.1, 61.6, 62, 62.3, and 62.5 °C, respectively. The M indicates the DNA molecular marker, including 100 bp,

300 bp, 500 bp, 700 bp (blod), 900 bp, and 1200 bp. C and T represent the control line and test line respectively.

Fig. S4 Optimization of the AS-PCR-LFA detection system. **A**: Optimization of the $MgSO_4$ in A256**T**. Number of 1-8 represent 0, 0.5, 1.0, 1.5, 2.0, 2.5, 3.0, and 3.5 mM, respectively; **B**: Optimization of primer concentration for A256**T**. Number of 1-8 represent final concentrations of 0.04, 0.1, 0.2, 0.3, 0.4, 0.5, 0.6, and 0.7 μM; **C**: Optimization of the number of PCR cycles for A256**T**. Number of 1-5 represent 15, 20, 25, 30, and 35 cycles, respectively; **D**: Optimization of the $MgSO_4$ in A551**T**; **E**: Optimization of primer concentration for A551**T**; **F**: Optimization of the number of PCR cycles for A551**T.** The M indicates the DNA molecular marker, including 100 bp, 300 bp, 500 bp, 700 bp (blod), 900 bp, and 1200 bp. C and T represent the control line and test line respectively.

Table S1 Selected and labeled primers for single nuclear polymorphisms detection in the *pfmdr1* gene.

Table S2 Optimized AS-PCR conditions for the *pfmdr1* gene.

Table S3 Comparison between nested PCR with sequencing and the AS-PCR-LFA platform for single nuclear polymorphisms detection in the *pfmdr1* gene with clinical isolates of *Plasmodium falciparum* parasites.

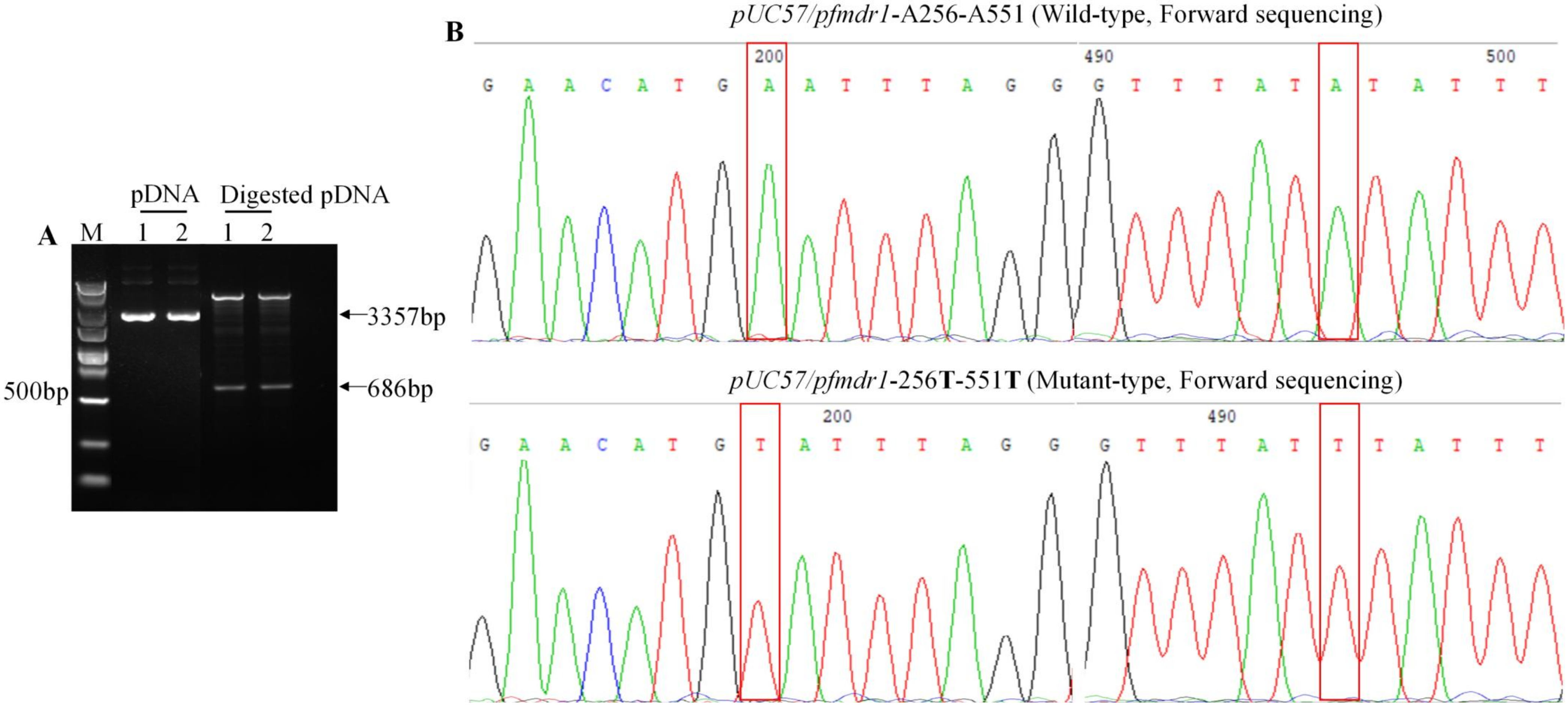
A
M
pDNA
1 2
Digested pDNA
1 2
500bp
3357bp
686bp
B
pUC57/pfmdr1-A256-A551 (Wild-type, Forward sequencing)
200
490
500
G A A C A T G A A T T T A G G G T T T A T A T A T T T
pUC57/pfmdr1-256T-551T (Mutant-type, Forward sequencing)
200
490
G A A C A T G T A T T T A G G G T T T A T T T A T T T

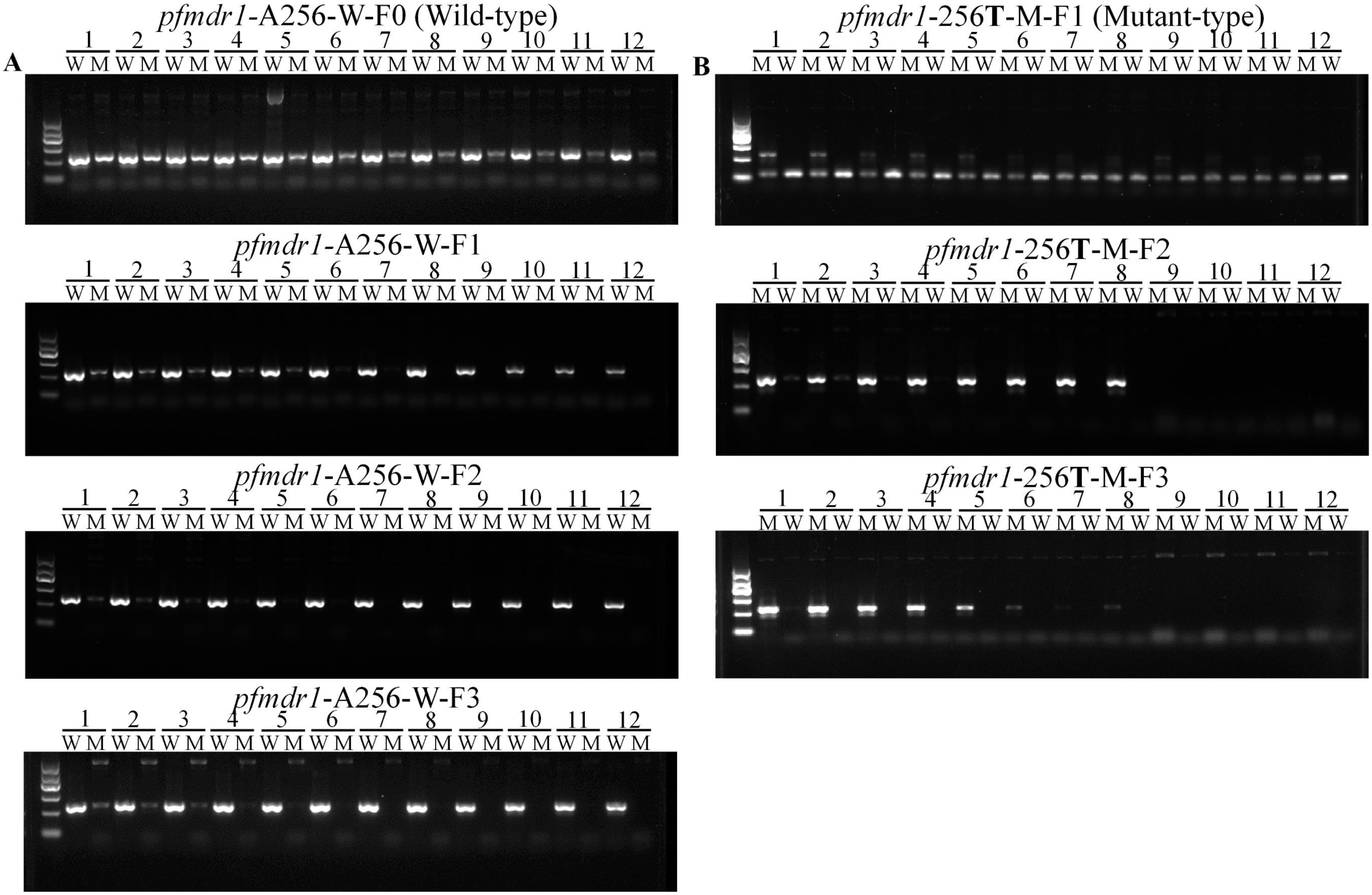

A
pfmdr1-A256-W-F0 (Wild-type)
1 2 3 4 5 6 7 8 9 10 11 12
W M W M W M W M W M W M W M W M W M W M W M W M
pfmdr1-A256-W-F1
1 2 3 4 5 6 7 8 9 10 11 12
W M W M W M W M W M W M W M W M W M W M W M W M
pfmdr1-A256-W-F2
1 2 3 4 5 6 7 8 9 10 11 12
W M W M W M W M W M W M W M W M W M W M W M W M
pfmdr1-A256-W-F3
1 2 3 4 5 6 7 8 9 10 11 12
W M W M W M W M W M W M W M W M W M W M W M W M
B
pfmdr1-256T-M-F1 (Mutant-type)
1 2 3 4 5 6 7 8 9 10 11 12
M W M W M W M W M W M W M W M W M W M W M W M W
pfmdr1-256T-M-F2
1 2 3 4 5 6 7 8 9 10 11 12
M W M W M W M W M W M W M W M W M W M W M W M W
pfmdr1-256T-M-F3
1 2 3 4 5 6 7 8 9 10 11 12
M W M W M W M W M W M W M W M W M W M W M W M W

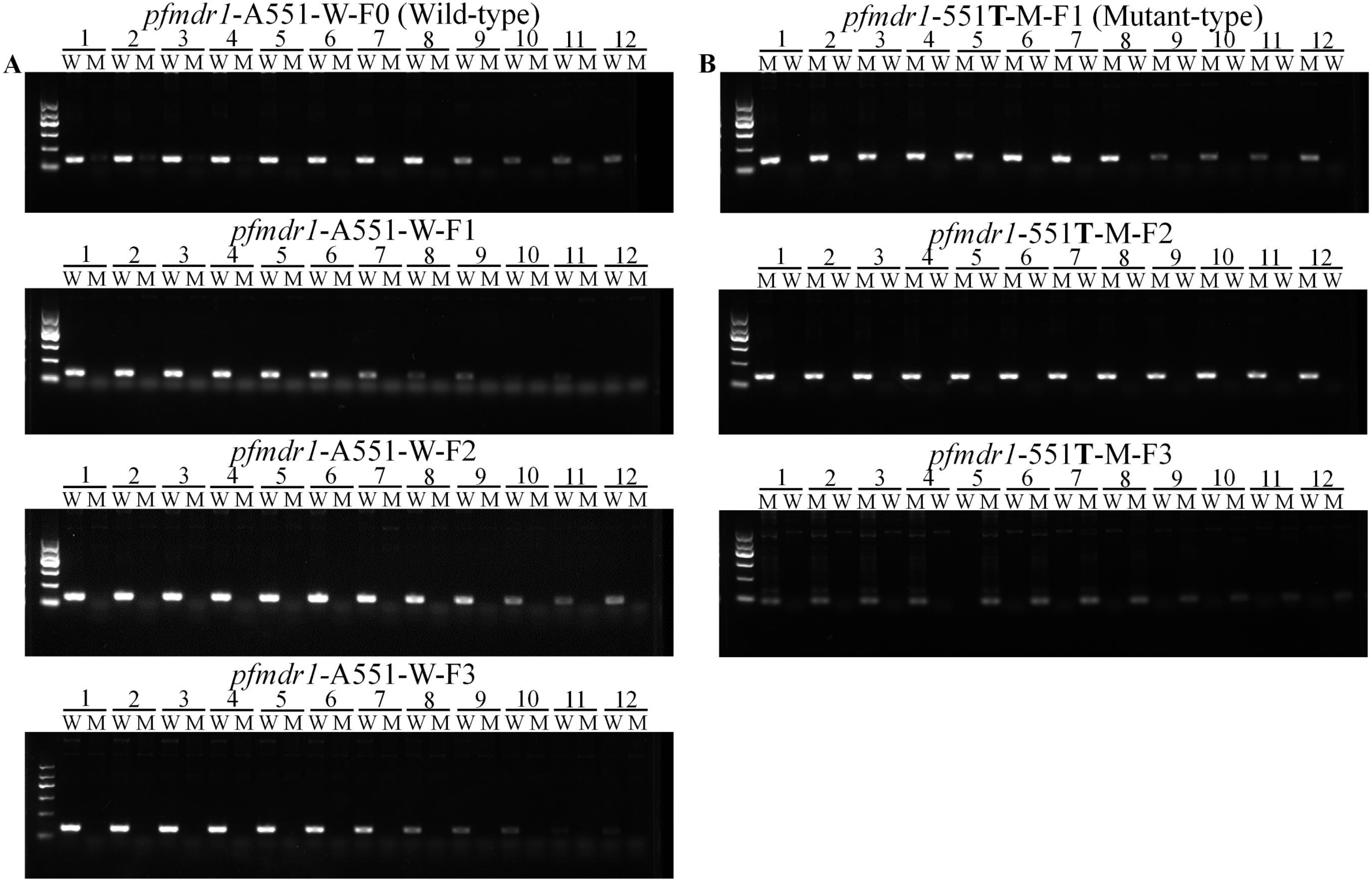
A
pfmdr1-A551-W-F0 (Wild-type)
1 2 3 4 5 6 7 8 9 10 11 12
W M W M W M W M W M W M W M W M W M W M W M W M
pfmdr1-A551-W-F1
1 2 3 4 5 6 7 8 9 10 11 12
W M W M W M W M W M W M W M W M W M W M W M W M
pfmdr1-A551-W-F2
1 2 3 4 5 6 7 8 9 10 11 12
W M W M W M W M W M W M W M W M W M W M W M W M
pfmdr1-A551-W-F3
1 2 3 4 5 6 7 8 9 10 11 12
W M W M W M W M W M W M W M W M W M W M W M W M
B
pfmdr1-551T-M-F1 (Mutant-type)
1 2 3 4 5 6 7 8 9 10 11 12
M W M W M W M W M W M W M W M W M W M W M W M W
pfmdr1-551T-M-F2
1 2 3 4 5 6 7 8 9 10 11 12
M W M W M W M W M W M W M W M W M W M W M W M W
pfmdr1-551T-M-F3
1 2 3 4 5 6 7 8 9 10 11 12
M W M W M W M W W M W M W M W M W M W M W M W M

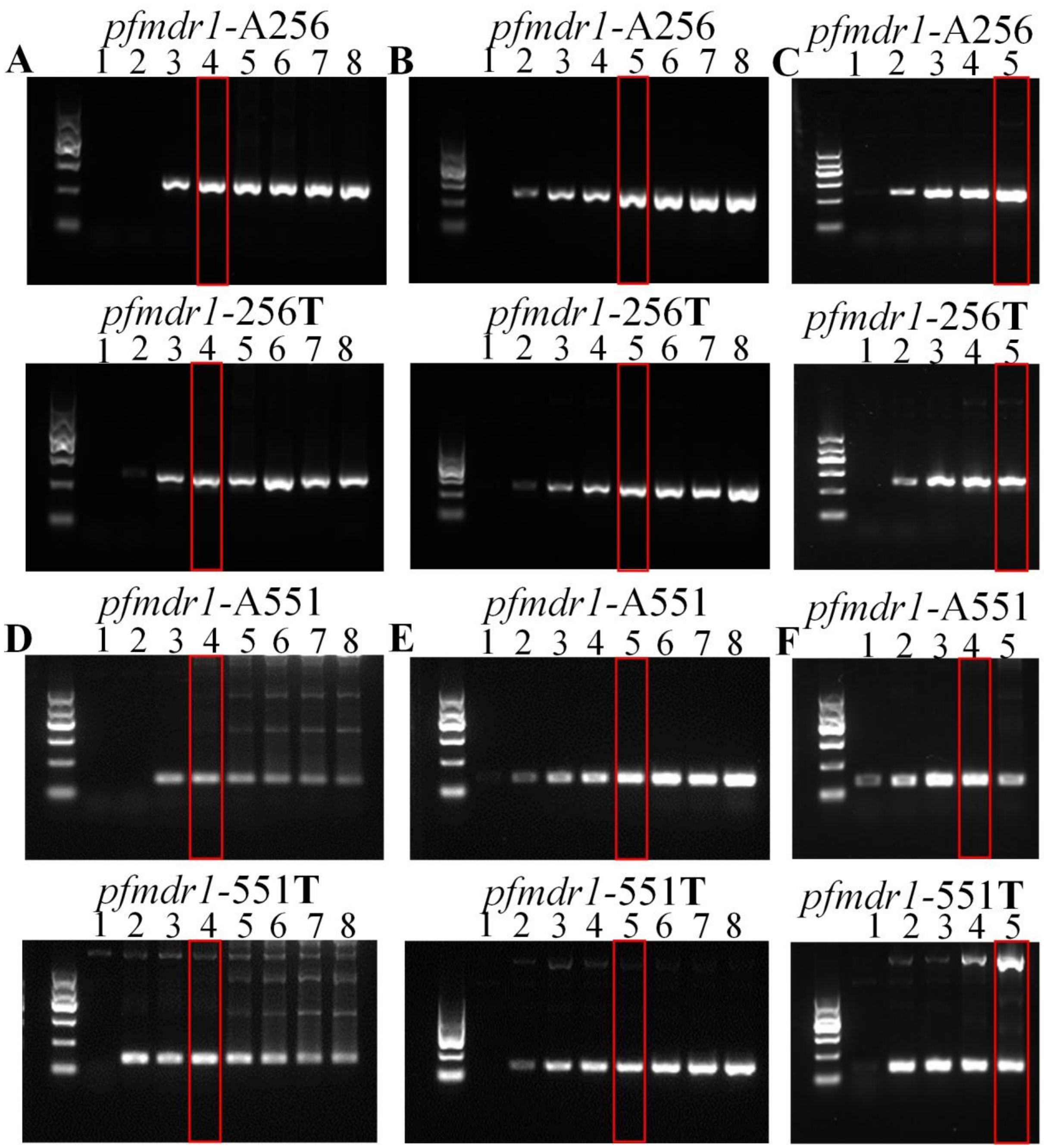
pfmdr1-A256
A
1 2 3 4 5 6 7 8
B
1 2 3 4 5 6 7 8
C
1 2 3 4 5
pfmdr1-256T
pfmdr1-A551
D
E
F
pfmdr1-551T
